# Unraveling the challenges in the diagnosis and management of desmoplastic fibroma of the mandible–a case report

**DOI:** 10.1186/s12903-024-04018-x

**Published:** 2024-02-20

**Authors:** Humayun Kaleem Siddiqui, Shahrukh Ali Khan, Anum Aijaz, Madiha Bilal Qureshi

**Affiliations:** 1 Oral & Maxillofacial Surgery, Dental Section, Department of Surgery, Aga Khan University Hospital, Karachi, Pakistan; 2 Dental Section, Department of Surgery, Aga Khan University Hospital, Karachi, Pakistan; 3 Pathology and Laboratory Medicine, Aga Khan University Hospital, Karachi, Pakistan

**Keywords:** Desmoplastic, Fibroma, Enucleation, Curettage, Bening

## Abstract

Desmoplastic fibroma (DF) is an uncommon bone tumor that originates from the mesenchymal tissue and despite being benign, exhibits aggressive behavior locally. The following report describes the case of a 7-year-old boy with a rapidly enlarging swelling on the right side of the mandible. After a thorough clinical examination, radiographic imaging, and histopathological analysis, the diagnosis of DF was confirmed. Treatment planning was formulated considering both the tumor’s tendency for local recurrence and the patient’s well-being. Due to the patient’s young age, segmental resection was not deemed appropriate, and an aggressive curettage and enucleation of the lesion followed by the bone graft was performed instead. The patient was kept under close follow-up for the first month of post-surgery and later reviewed after 3, 6, 9, and 12 months, respectively. Good bone healing was observed on radiographs. The patient did not show any signs of recurrence based on clinical or radiographic assessments and did not exhibit any neurosensory deficits as well.

## Introduction

Desmoplastic fibroma (DF) is an uncommon intermediate, locally aggressive bone tumor that does not metastasize to other sites of the body. It is known to cause significant bone destruction [1, 2] with a high recurrence rate following surgical removal [3]. Jaffe originally described an aggressive fibromatosis of the femur, tibia, and scapula that was largely osseous in origin in 1958 [4]. The incidence of this tumor is less than 1% compared to other tumors of the bone [5, 6]. Desmoplastic Fibroma has the potential to impact any bone in the body, it is frequently observed in the mandible (22%), followed by the femur (15%), pelvic bones (13%), radius (12%), and tibia (9%) [3, 7].

Histologically, it mimics its’ soft tissue counterpart, the “Desmoid Tumor.” The name “Desmoid” comes from the Greek word “demos,” which means band or ligament, and was first described by Johannes Müller, a German physiologist and anatomist in 1838 [8]. In 1965, Griffith and Irby were the first to present a report on a desmoplastic fibroma of bone [9], and ever since, numerous studies on DF have been reported [2, 3, 10–15]. The exact etiologic factors for DF are presently not known but possible associations with trauma [4], endocrine factors, [4, 16] and genetic abnormalities [17] have been proposed.

When present in the jaw bones, mandible, and maxilla account for about 86% and 14% of cases respectively. In either jaw, there is a preference for the posterior location. Both genders are equally affected [15]. On average, patients receive the final diagnosis of DF at 15.1 years of age [18]. The symptoms of DF are usually non-specific with patients experiencing a generalized discomfort over the affected region and, sporadically, a palpable mass. Additionally, DF may be detected incidentally during medical examinations [19].

World Health Organization (WHO) characterizes it as an intermediate, locally aggressive tumor based on its low to moderate cellularity and lack of pleomorphism/mitoses [20]. Clinical characteristics and histologic features distinguish DF from other types of fibroblastic tumors, which generally are less aggressive in nature. Desmoplastic fibroma is considered non-odontogenic fibromatosis in the jawbone [1, 21–23].

This article aims to report the challenges encountered in making an accurate diagnosis and surgical management of desmoplastic fibroma of the mandible in a young boy since the clinical appearance and radiologic features were more indicative of a more aggressive tumor. This impression affected the surgical approach to the tumor, and several treatment regimens with various results were used. The treatment was performed at the Oral and Maxillofacial Surgery Department of Aga Khan University Hospital, Karachi, Pakistan.

## Case report

### Initial presentation

A six-year-old boy with no comorbid visited the outpatient clinic at the Oral and Maxillofacial Surgery Department, AKUH in May 2022. The boy reported swelling on the right side of his face that had grown rapidly over a period of 2 months leading to facial asymmetry. There was no significant medical history and no previous history of trauma.

**Fig. 1:**
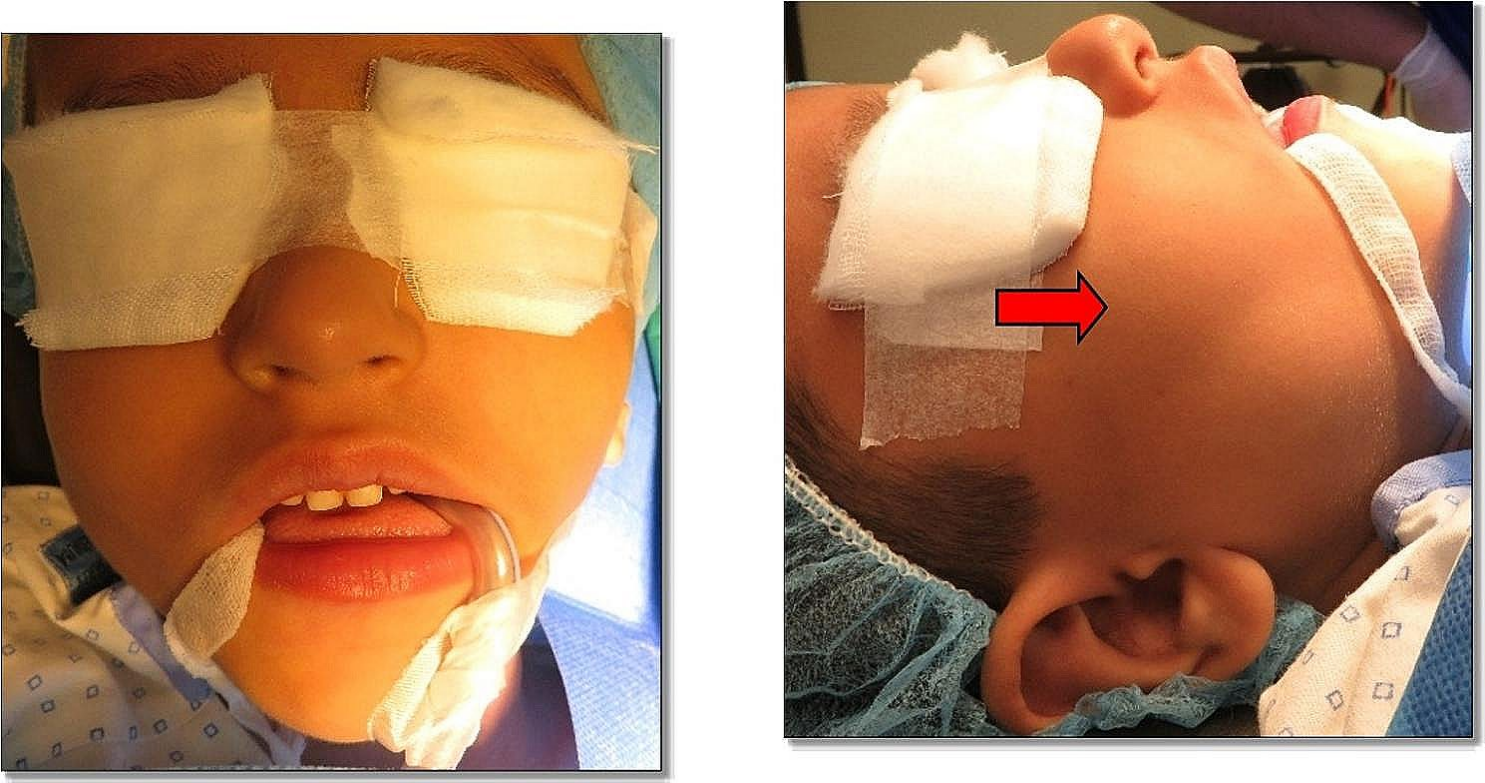
(a, b) Extraoral images showing swelling of right side of the lower jaw, leading to facial asymmetry

**Fig. 2:**
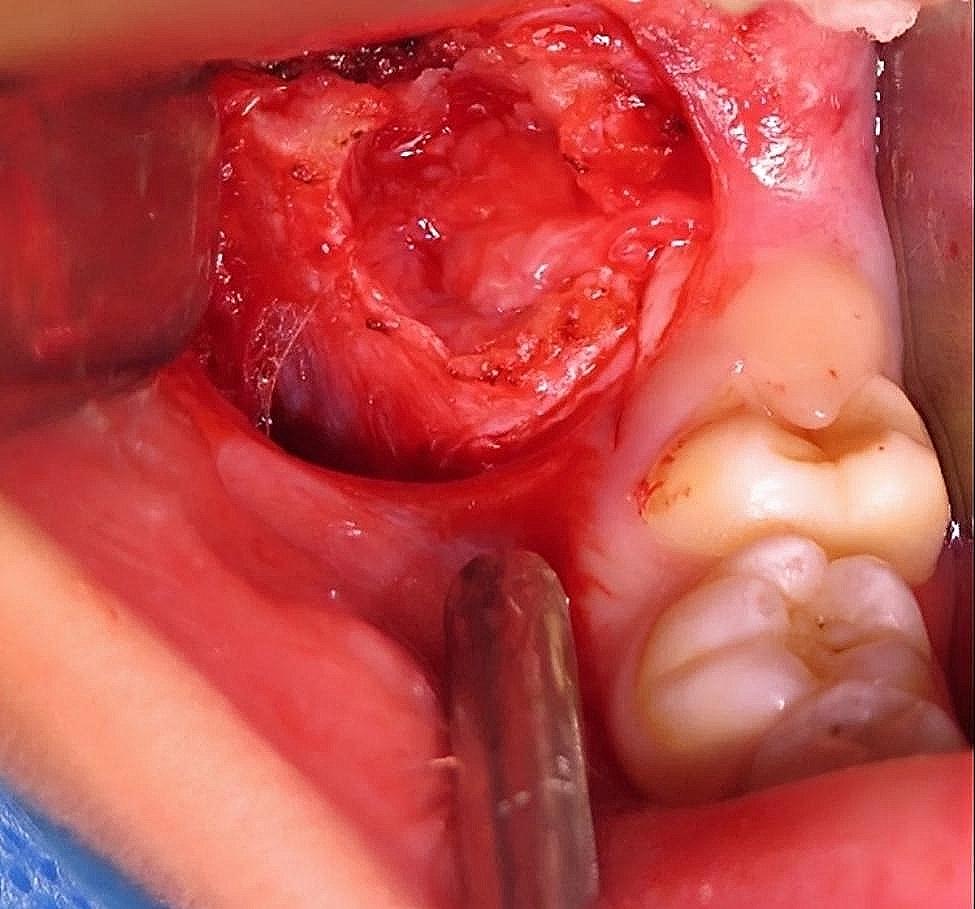
Intra oral image showing obliteration of right buccal vestibule in relation to partially erupted 46

On examination, the swelling was noted to be firm, diffuse, and non-tender on palpation extra-orally. No signs and symptoms of TMJ dysfunction or regional lymphadenopathy were observed. Intra orally, the swelling was extending distal to the first lower right permanent molar and towards the ascending ramus anteroposteriorly. The overlying mucosa appeared normal. Oral hygiene was fair, and there were no signs of carious lesions. A complete blood count (CBC) showed a high eosinophilic count. Clinical differential diagnoses included ameloblastoma and adenomatoid odontogenic tumor. The extra and intraoral features are shown in Figs. 1 and 2.

### Radiological investigations

**Fig. 3:**
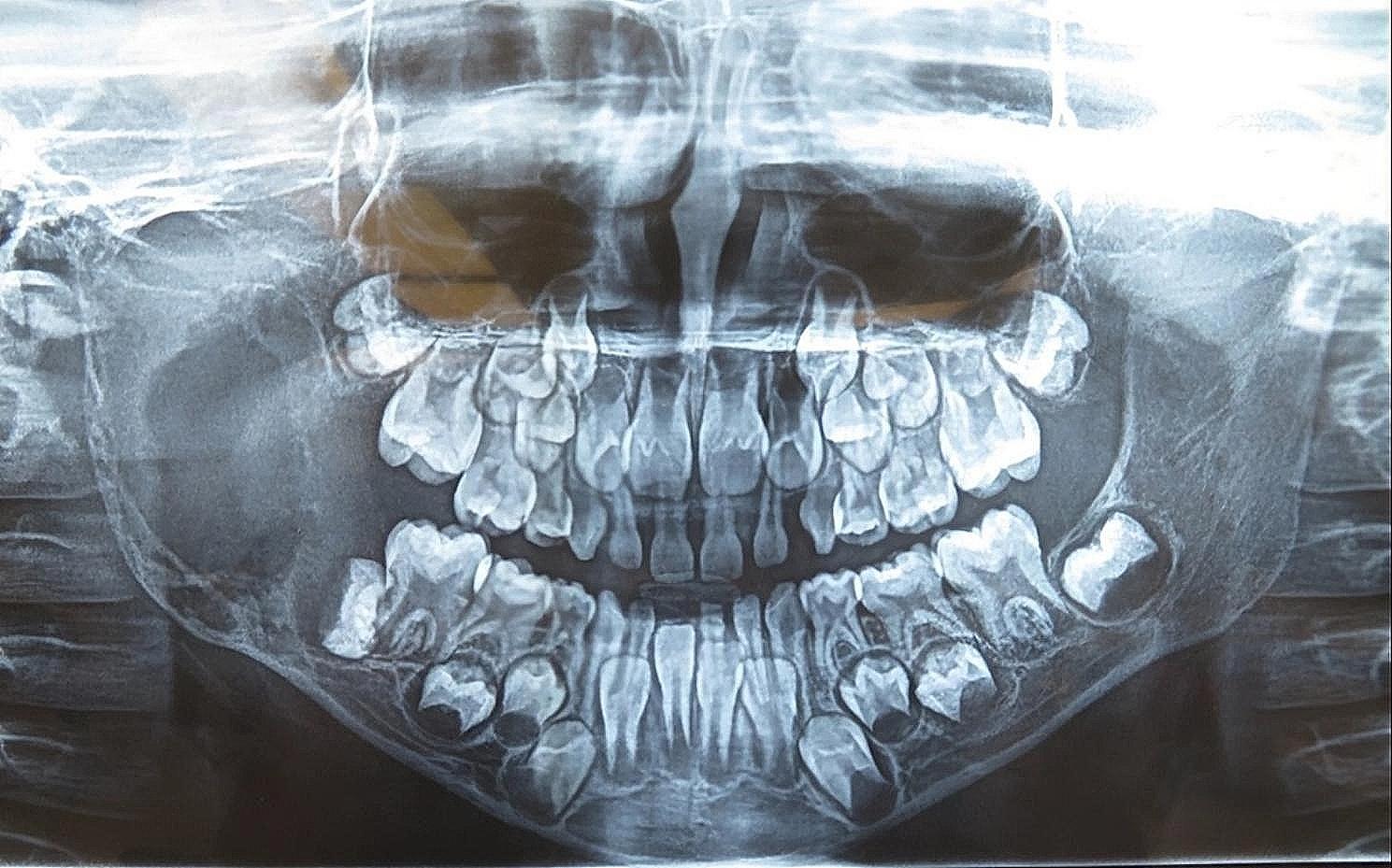
Preoperative OPG showing well defined radiolucency on the right side of the ramus of the mandible with egg shell thin inferior border, without any pathological fracture and tooth displacement

**Fig. 4:**
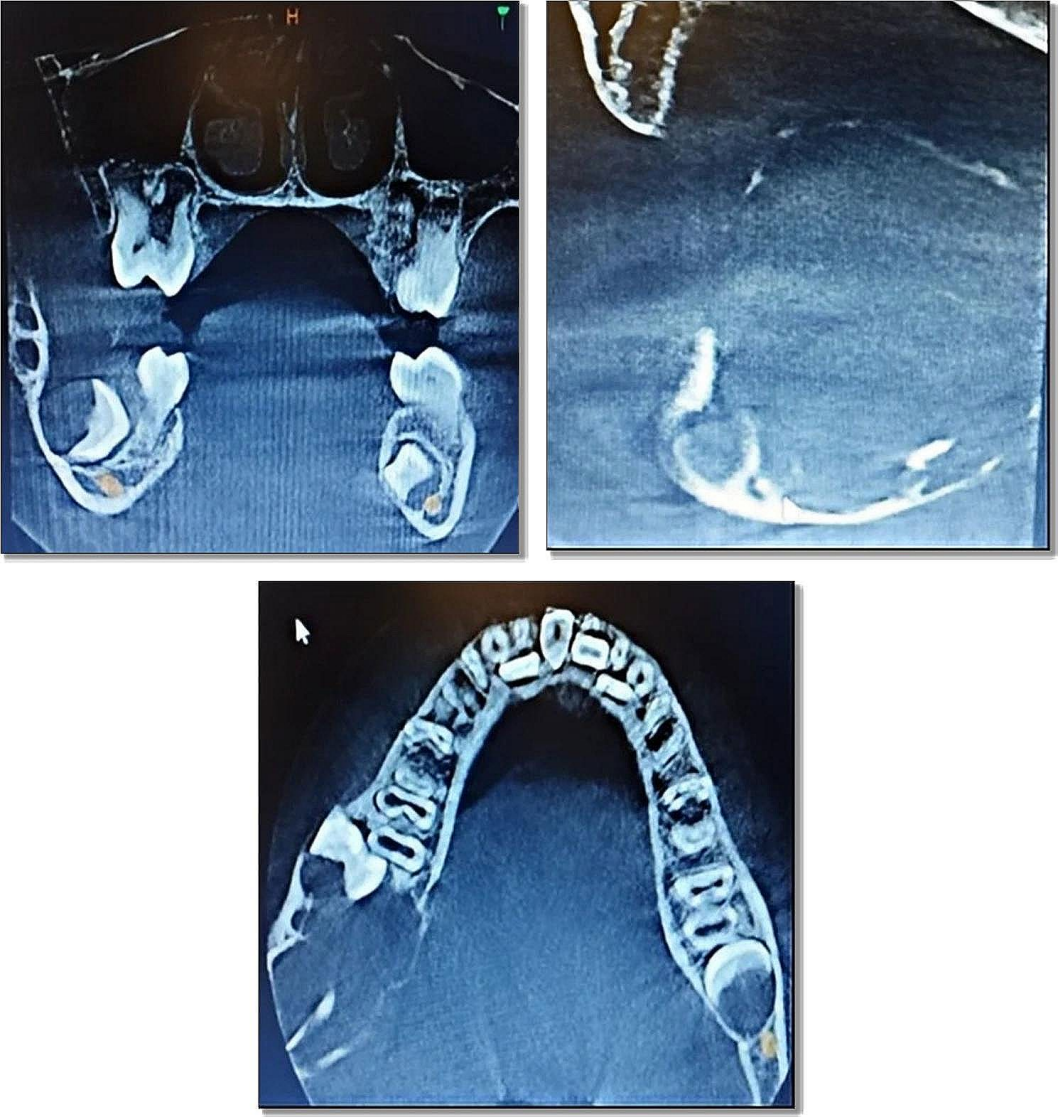
CT scan showing the extension of lesion all three planes ; Note that the lesion has eroded the cortex and is expanding into the soft tissue

On a routine orthopantomogram, a well-defined multilocular radiolucent area on the ascending ramus of the mandible was observed on the right side. The cortical bone appeared eggshell thin on the inferior border of the mandible but with no evidence of pathological fracture, as shown in Fig. 3. A Computed Tomography (CT) scan with contrast revealed a lesion with well-defined borders containing internal soft tissue in the ramus of the mandible with peri coronal involvement of the lower right second molar tooth measuring about 32 × 32 × 33 mm in size. Cortical bone expansion and bowing were evident without any significant displacement of internal structures or periosteal reaction. The extent of the lesion on the sagittal, coronal, and axial planes is shown in Fig. 4.

### Histopathology

**Fig. 5:**
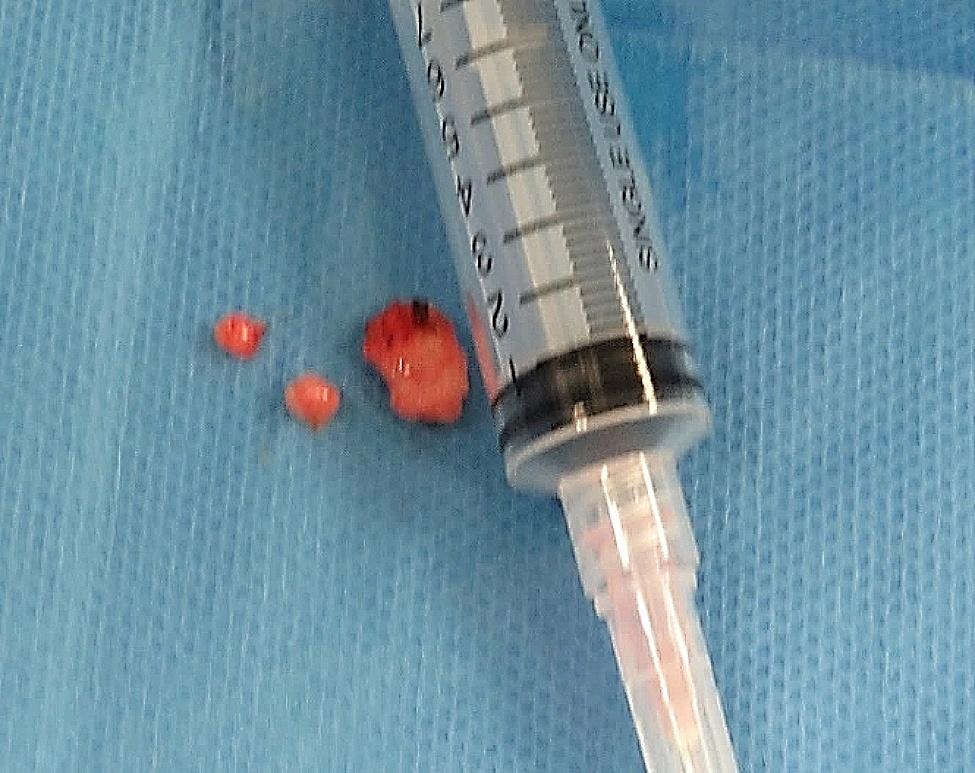
Specimen taken for initial biopsy consisting of 3 tan-white irregular tissue pieces from right ramus of the mandible

Initially, a fine needle aspiration biopsy (FNAC) was advised, but inconclusive. Later, an incisional biopsy was performed under general anesthesia. The specimen was coded as “Intra-bony tissue from the right ramus of mandible” and consisted of 3 tan-white irregular tissue pieces that measured 1.5 × 1 cm in aggregate, as shown in Fig. 5.

**Fig. 6:**
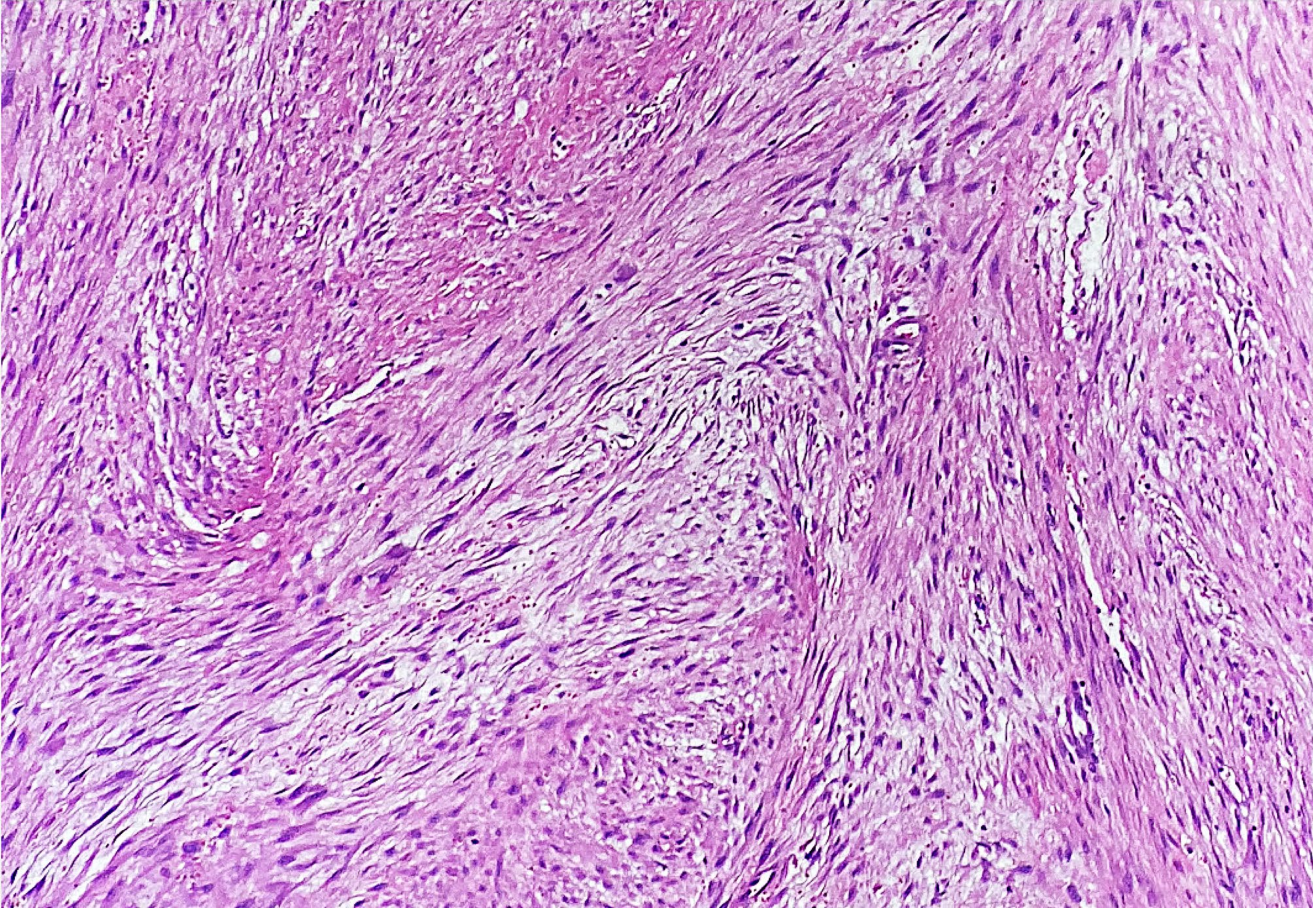
Long sweeping fascicles of fibroblasts/myofibroblasts in a collagenous background. H&E, 100X

Histology showed a moderately cellular neoplastic lesion composed of long sweeping fascicles of spindle-shaped cells (Fig. 6). The neoplastic cells had elongated, oval vesicular nuclei, pinpoint nucleoli, and moderately eosinophilic cytoplasm. The background was collagenous to focally myxoid, and prominent extravasation of red blood cells was present. Scattered small to intermediate sized ectatic blood vessels and occasional multinucleated giant cells were seen. Up to 2–3 mitoses/10 high power fields (HPFs) were noted without any necrosis or atypia. Differential diagnoses based on histology included desmoplastic fibroma, nodular fasciitis, low-grade central osteosarcoma, and low-grade myofibroblastic sarcoma.

Nodular fasciitis and low-grade myofibroblastic sarcoma typically affect young adults, and intraosseous location is very rare. Low-grade central osteosarcoma shows mild to moderate atypia in the spindle cells with focal osteoid formation, which was absent in this case.

### Immunohistochemical staining

Immunohistochemical stains (IHC) showed patchy positivity for Anti-Smooth Muscle Actin (ASMA), and Beta-catenin, whereas immunostains CD10, Desmin, SATB2, CD34, and S100 were negative.

### Definitive diagnosis

After evaluating clinical, radiographic, and histological features, the tumor was best diagnosed as Desmoplastic fibroma.

### Surgical procedure

**Fig. 7:**
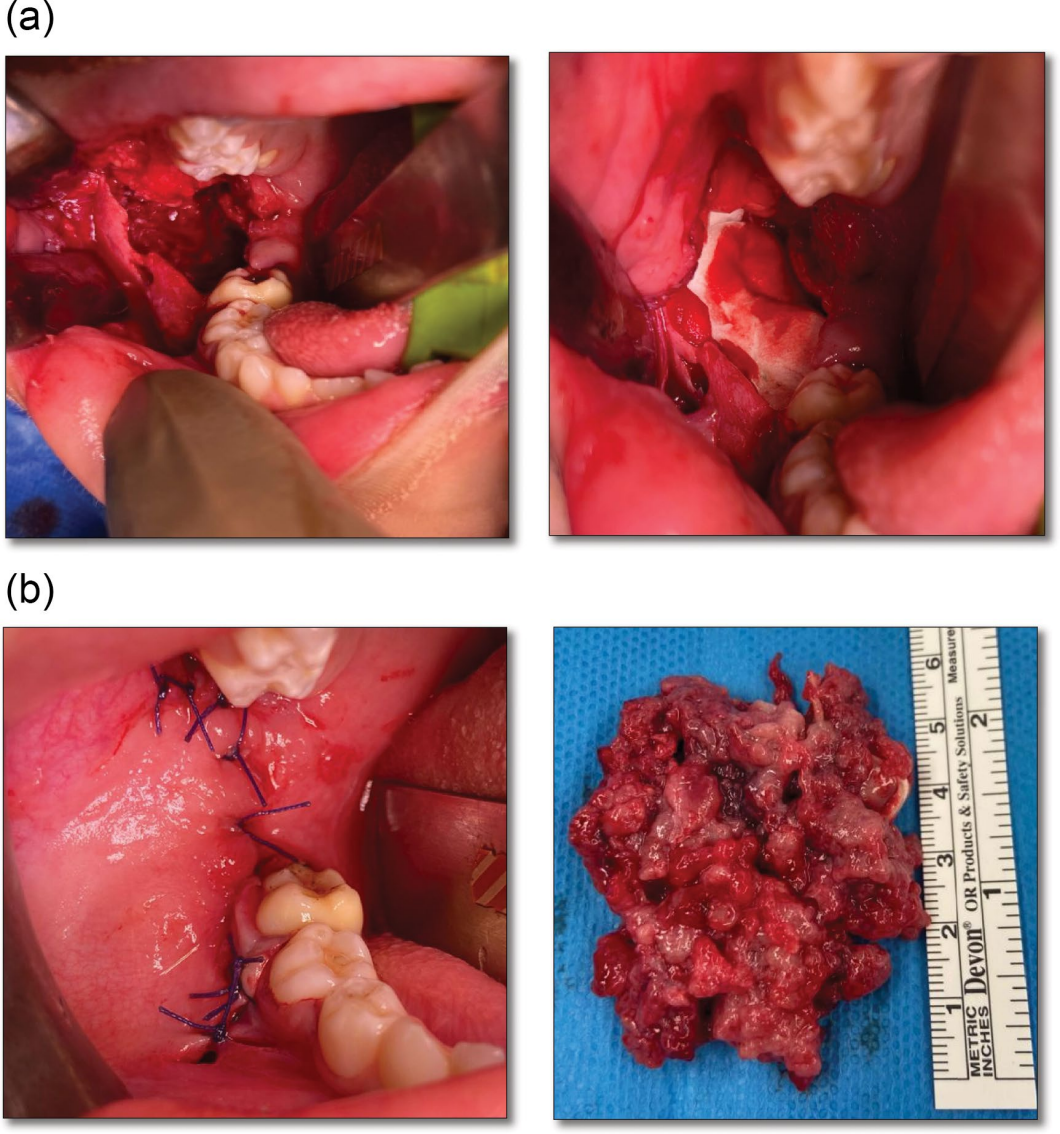
(a) Intraoral approach through ramus incision. (b) Placement of allogenic bone graft. (c) Secondary healing through resorbale interrupted sutures. (d) Enucleated lesion consisting of multiple tan-brown irregualr tissue pieces and embedded deciduous tooth

The case was taken to the tumor board, and it was decided to go for complete enucleation of the lesion, followed by placement of an allogenic bone graft instead of resection of the mandible under general anesthesia (Fig. 7). The higher risk of recurrence was communicated to the patient’s family well before the surgery.

Following aseptic conditions under general anesthesia, the posterior mandible was approached intraorally through a ramus incision. The tumor had minimal bleeding and was removed in pieces. The inferior alveolar vascular bundle, along with the developing dentition, was identified and protected. The tumor was removed until the bony boundaries. Although it was extremely thin in the angle area, no fracture or deviation was observed in intra and postoperative visits. After removal of the tumour allogenic bone graft was placed to fill in the cavity. The incision was closed with resorbable sutures in an interrupted fashion. The Patient’s parents were advised to give a semisolid diet for the next 4 weeks and report any deviation of mouth observed. The patient was released the same day after an uneventful post-operative phase. The diagnosis was the same as in the initial biopsy.

### Follow-ups

**Fig. 8:**
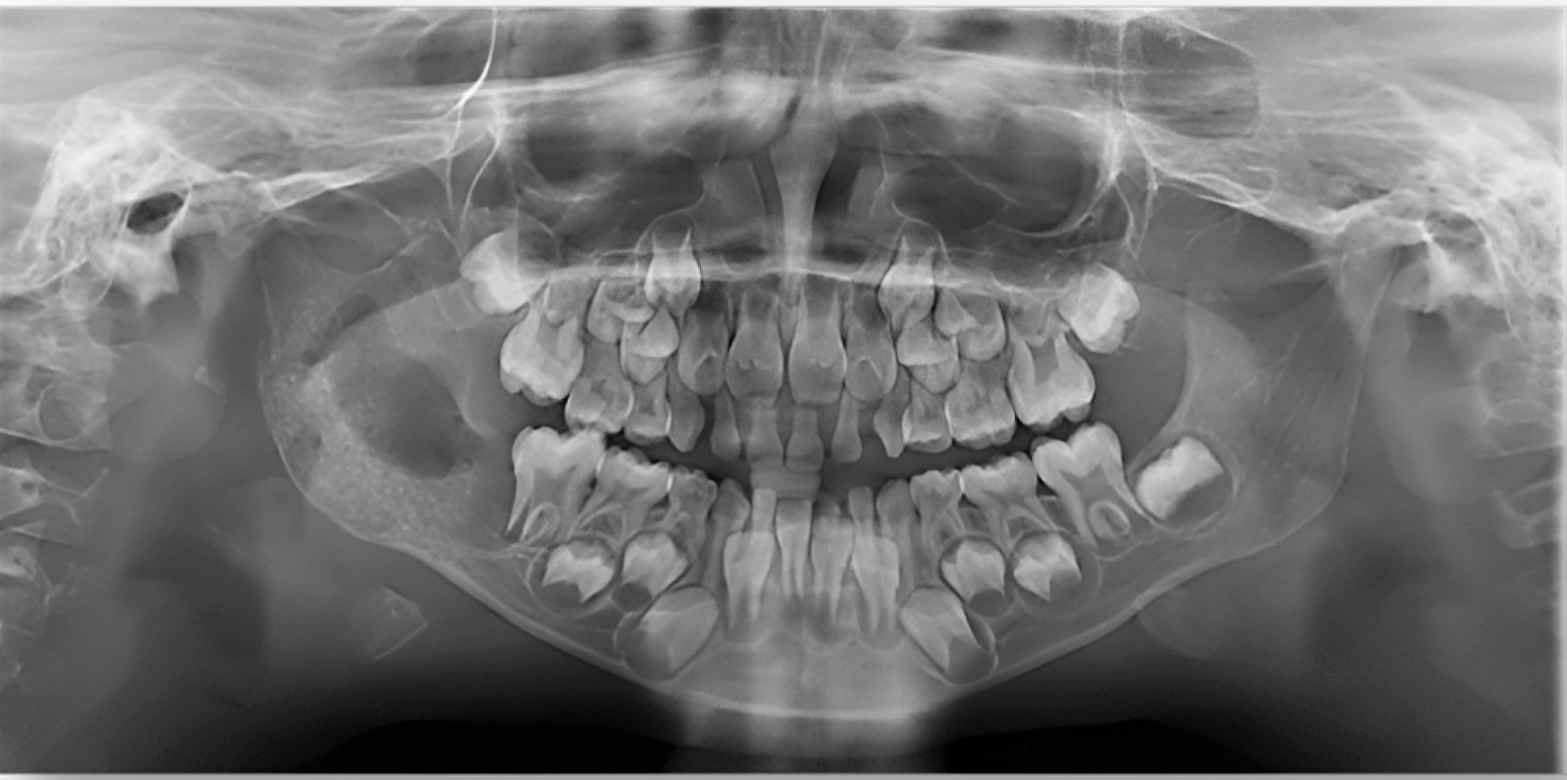
Postoperative OPG at 2 weeks follow up showing satisfactory bone remodeling

**Fig. 9:**
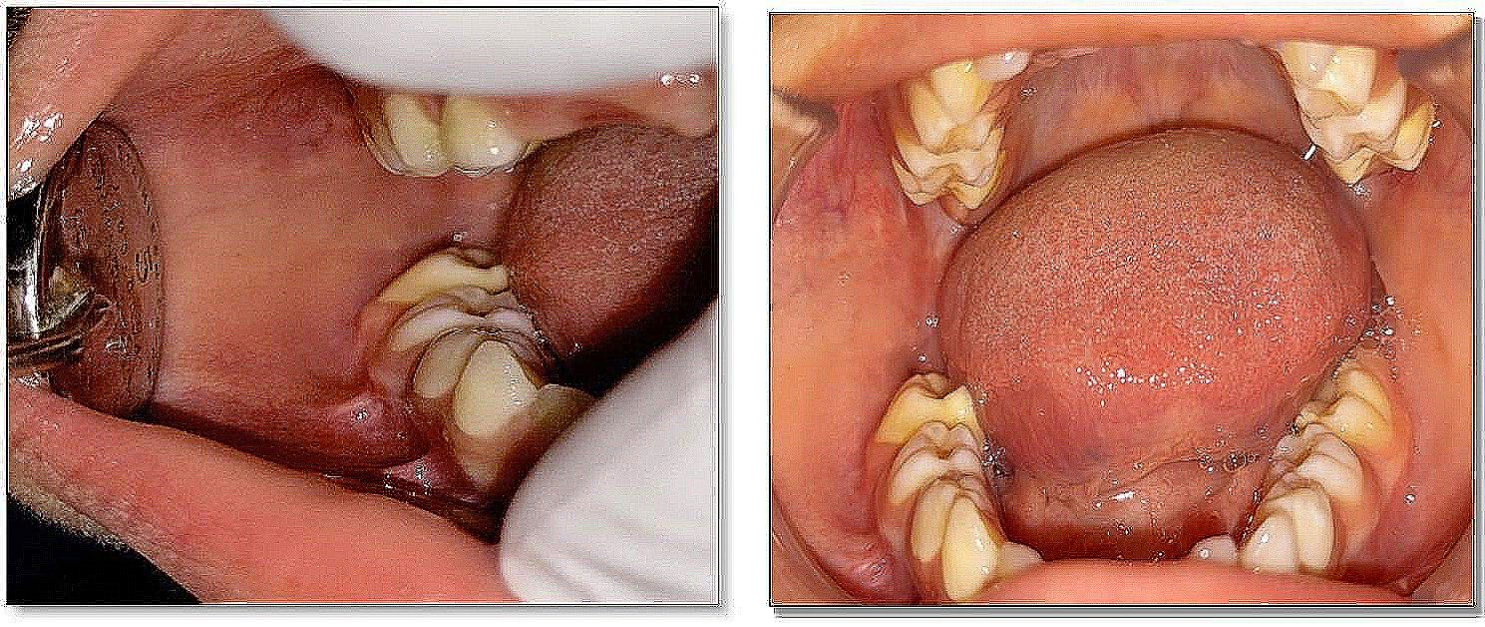
Intraoral images at 3 months post-operative follow up showing satisfactory soft tissue healing

**Fig. 10:**
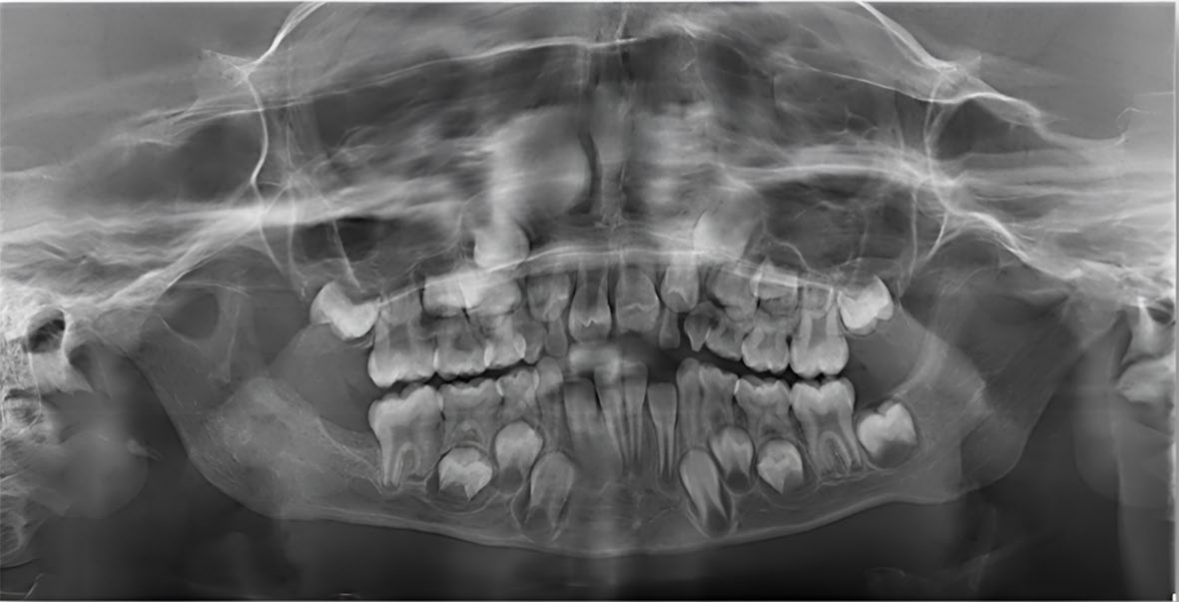
Post operative OPG at 12th month of the follow up demonstrating significant and continued bone remodeling, indicating successful graft integration and enhanced bone density at the grafted site

**Fig. 11:**
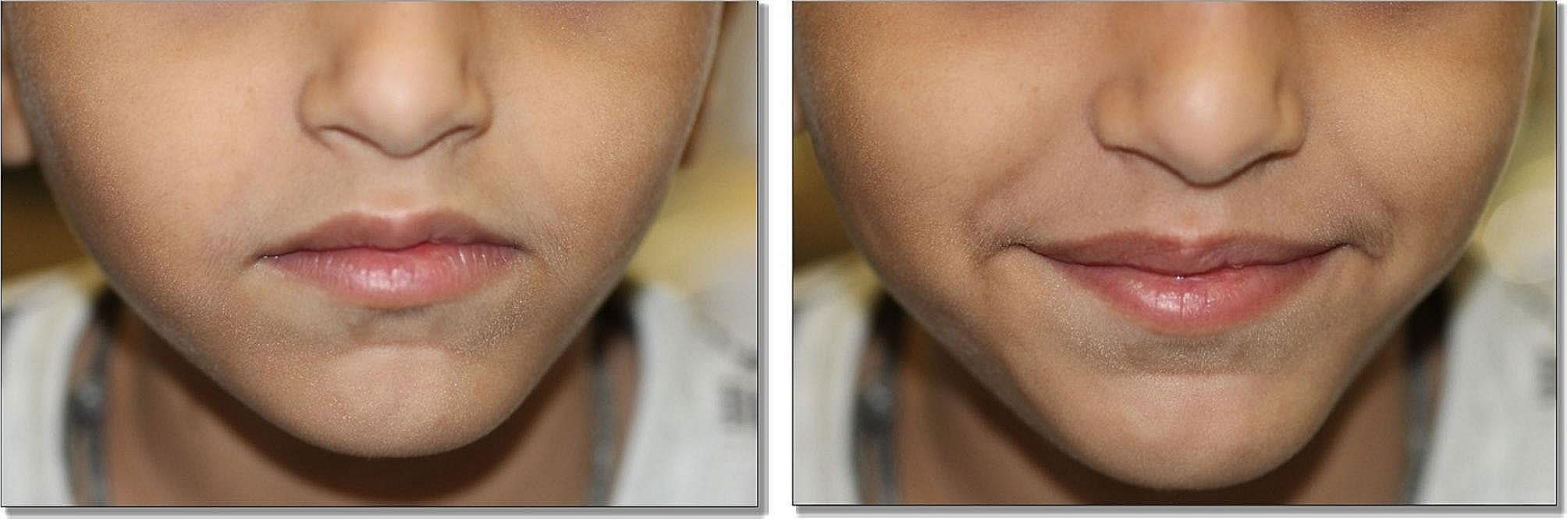
Extraoral image at 16 months post-operative follow up showing satisfactory facial appearance on smiling and relaxed state

The patient was kept under close follow-up for the first month of post-surgery and later reviewed after 3, 6, 9, and 12 months, respectively (Figs. 8, 9, 10 and 11). Good bone healing was observed on radiographs. The patient did not show any signs of recurrence based on clinical or radiographic assessments and did not exhibit any neurosensory deficits as well.

## Discussion

DF affecting the jaws is typically detected in individuals in their second and third decades of life, with an average age of 15.7 years and a slight inclination towards females [24]. However, in this case, the patient was a 7-year-old boy. Although DF may affect any bone throughout the body, the mandibular molar-ramus-angle area is the most commonly affected side in the maxillofacial region [25], which is consistent with the location observed in our case report. The diagnosis of DF requires the correlation of clinical, radiographic, and histopathological data [26].

The signs and symptoms are typically non-pathognomonic and develop gradually, with significant damaging lesions frequently found during the initial presentation. The two most frequently encountered clinical features are painless mandibular swelling and restricted mandibular opening resulting from the lesion’s locally aggressive and expansile nature, which causes it to invade the surrounding soft tissues [27, 28]. The chief complaints in our patient were discomfort and swelling, and there was also noticeable asymmetry, but we did not observe any pathological fracture or tooth displacement.

The most commonly reported radiographic features in the literature for DF are clearly defined non-sclerotic marginated lytic lesions (94%), internal pseudo trabeculation (91%), and bone expansion (89%) [28, 29] which are similar to our case. This rare lesion, as per radiological concepts has the potential to resemble other fibro-osseous, benign, and especially malignant lesions, including ameloblastoma, aneurysmal bone cyst, odontogenic myxoma, central hemangioma, chondromyxoid fibroma, eosinophilic granuloma [30–35] and hence should be considered in the differential diagnosis. Therefore, relying solely on imaging studies to establish a diagnosis can be challenging. In cases, where there is suspicion of cortical damage, it is recommended to undergo a CT scan as it can enhance the clarity of bone structure and precisely measure the extent of cortical bone destruction [36]. Magnetic resonance imaging is preferred if extraosseous tumor growth is suspected and for surgical planning due to its ability to distinguish intraosseous tumors from normal bone marrow but does not significantly contribute to the differential diagnosis [37, 38]. Desmoplastic fibroma of bone can resemble more malignant pathologies, such as fibrosarcoma, intra-osseous osteosarcoma, and metastases [20, 39] if cortical destruction and a soft tissue mass are present.

Histological analysis is considered the gold standard for diagnosis [26]. WHO describes the classic histologic features of desmoplastic fibroma: characterized by long sweeping fascicles of slender, spindle to stellate cells with minimal cytological atypia and a significant amount of background collagenous matrix [20]. The cells contain vesicular nuclei with pinpoint nucleoli. The lesion is marked by low cellularity with occasional hypercellular foci as well as a lack of a capsule and an infiltrative nature [25]. The tumor shows the positive expression of immunomarkers Smooth Muscle Actin, Vimentin, and Beta-catenin, while SATB2 is typically negative. To rule out other tumors in the clinical differentials, a histopathological examination is necessary. For example: distinguishing DF from low-grade fibrosarcoma and low-grade intraosseous osteosarcoma can be quite challenging. Low-grade fibrosarcoma typically shows a herringbone pattern with prominent atypia and mitoses. Low-grade intraosseous osteosarcoma may show mild to moderate atypia and osteoid formation may be sparse [25]. When taking a biopsy of such lesions, it is recommended to avoid samples from the periphery since native/reactive bone at the periphery may be misinterpreted as an osseous component of a fibro-osseous bone tumor or osteosarcoma [40]. 

For DF, several treatment strategies have been utilized, including surgery, radiation therapy [9, 13], and chemotherapy either alone or in conjunction with other procedures [41]. Radiation is not advised due to its low success rate and the possibility of post-radiation sarcoma as a result of its mutagenic effects [28]. Iwai et al. [42] stated that patients who underwent resection or wide excision did not experience any recurrence, whereas those who underwent simple excision or enucleation had a recurrence rate ranging from 20 to 40%. While patients who underwent curettage alone had a significantly higher recurrence rate of 70%. This highlights the importance of distinguishing DF from other bone lesions because of its high recurrence and aggressive behavior that significantly impacts surgical therapy.

In the present case, segmental resection was not chosen as a treatment option because of the patient’s young age. Instead, aggressive curettage/enucleation of the lesion followed by placement of a bone graft was preferred to preserve the architecture of the mandible. The patient has not displayed any clinical or radiographic recurrence throughout the 1-year follow-up evaluation, but given the high recurrence rates of DF, it’s essential to maintain monitoring, as a minimum follow-up period of three years is currently considered necessary to identify any potential recurrence of DF [17]. Therefore, the patient will undergo follow-up assessments at both the 2-year and 3-year to ensure a thorough evaluation of recurrence risk and treatment outcomes.

## Conclusions

Desmoplastic fibroma (DF) is an uncommon intermediate, locally aggressive bone tumor that does not metastasize to other sites of the body. The diagnosis of DF requires clinical, radiographic, and histopathological correlation. Treatment planning for DF must be carefully formulated, taking into account the tumor’s tendency for local recurrence and the patient’s well-being. The higher risk of recurrence has to be communicated to the patient well before the surgery.

## Data Availability

The datasets used and/or analysed during the current study available from the corresponding author on reasonable request.
